# Supplementary material for: Exogenous melatonin promotes seed germination and osmotic regulation under salt stress in cotton (Gossypium hirsutum L.)
Source: PLoS One. 2020 Jan 31;15(1):e0228241. doi: 10.1371/journal.pone.0228241 (PMC6994006; doi:10.1371/journal.pone.0228241)
Supplement: Attachment — Submitted filename: Response to Reviewers.docx [file pone.0228241.s003.docx]

Dear Sergey Shabala,

On behalf of my co-authors, we thank you very much for giving us an opportunity to revise our manuscript, we appreciate editor and Reviewers very much for their positive and constructive comments and suggestions on our manuscript entitled “Exogenous melatonin promotes seed germination and osmotic regulation under salt stress in cotton (*Gossypium hirsutum* L.) (submitted as PONE-D-19-18506)”. Those comments are all valuable and very helpful for revising and improving our paper, as well as the important guiding significance to us researches. We have studied comments carefully and have made correction which we hope meet with approval. Revised portion are marked in red in the paper.

The main corrections in the paper and the responds to the Reviewer’s comments are as flowing:

Responds to the Reviewer’s comments:

**Reviewer #1**

**Major comments**

1. According to the significance letters on Figure 1, no significant difference of germination rate was found between salt stress cotton plants with and without the melatonin treatment. Although it suggest a difference, an overlap of significance letters (for example, b and bc, or a and ab) between different groups indicates no significant difference at 0.05 level. Since only three replicates are conducted in this study, more replicates could help to improve the data accuracy. Or authors may redo the statistical analysis.

**Response:** We have made correction according to the Reviewer’s comments. We have add three replicates and redo the statistical analysis.

2. More in depth discussion will improve the quality of the current manuscript. For example, how melatonin reduced ROS content and maintained better K^+^/Na^+^ ratio in cotton seeds under salinity? Is it a direct and transporters? Or through reduce ROS content, melatonin indirectly affects ion transport.

**Response:** We have made correction according to the Reviewer’s comments. Part of the discussion was added.

3. Three replicates are the minimum requirement of biological studies. More replicates could improve the accuracy of data.

**Response:** We have made correction according to the Reviewer’s comments. Part of replicates was added and redo the statistical analysis.

**Minor comments**

4. Ln35: “the first evidence” is a bit over claim. For me, “evidence” is more related to something are clearly understood. Consider to revise it.

**Response:** We have made correction according to the Reviewer’s comments.

5. L80-81: Cite (Mattuis 2014 J Exp Bot; Wu 2018, Crop J)

**Response:** We have made correction according to the Reviewer’s comments. We have added reference “Wu 2018, Crop J”. However, I am sorry that “Mattuis 2014 J Exp Bot” reference was not found.

6. Ln330-331: Consider to rewrite it.

**Response:** We have made correction according to the Reviewer’s comments.	`

7. Ln350: Consider to revise it. I would say “Na^+^ toxicity is one of the main component of salt stress in plants.”

**Response:** We have made correction according to the Reviewer’s comments.

8. Ln396: I would use “impaired” rather than “restricted”.

**Response:** We have made correction according to the Reviewer’s comments.

9. Ln396-397: Remove “Furthermore”.

**Response:** We have made correction according to the Reviewer’s comments.

10. Ln410: “affected” was repeated.

**Response:** We have made correction according to the Reviewer’s comments.

11. Ln427-428: “internal and external balance of cells”. It is not clear. Consider to revise or remove it.

**Response:** We have made correction according to the Reviewer’s comments. We have deleted the relevant it.

12. Ln429: Over claim. No direct evidence showed in the current manuscript to support the claim that melatonin can directly scavenge ROS.

**Response:** We have made correction according to the Reviewer’s comments.

13. Ln459: “high K^+^ and low Na^+^” is not accurate. Under salt stress, Na^+^ accumulation and K^+^ loss happened. Maybe “to maintain K^+^/Na^+^ ratio” will be more accurate.

**Response:** We have made correction according to the Reviewer’s comments.

**Figures**

14. For better reading experience, according to the real setting of treatments described in the current manuscript, I would suggest to change “CK1” to “Con” or “Ctrl”, “CK_2_” to “NaCl”, “MT10” to “MT10 + NaCl”, “MT20” to “MT20 + NaCl”, “MT50” to “MT50 + NaCl”, and “MT100” to “MT100 + NaCl”.

**Response:** We have made correction according to the Reviewer’s comments. Thank you for your suggestion.

15. Figure 2. The orders of significance labels are not appropriately presented. For example, in day2, “CK2” is the highest and is labelled with significance letter a, then the significance letter d should be assigned to the lowest one which is the “MT20” group. Also, please double check the assigned letters. Given the small error bar, “MT20” and “MT50” seem to have a significant difference.

**Response:** We have made correction according to the Reviewer’s comments and redo the statistical analysis.

16. Figure 5a. Why Na^+^ content in “CK2” group (150 mM NaCl) is decreased overtime? Also, in day 6, “MT50” showed no protective role in preventing Na^+^ accumulation in cotton seeds under salinity.

**Response:** We have made correction according to the Reviewer’s comments. We recheck the statistical analysis and add three replicates, redo the statistical analysis.

17. Figure 5c. Please recheck the statistical analysis. Given the big error bars, most of the treatment showed no significant difference of K^+^ content. Another way is to increase sample replicates since only three replicates are conducted in the current manuscript.

**Response:** We have made correction according to the Reviewer’s comments. We recheck the statistical analysis and add three replicates, redo the statistical analysis.

**Reviewer #2**

**Introduction**

18. Ln57-59: there are relevant melatonin data and references, which should be included. Melatonin was introduced through cyanobacteria that became chloroplasts and mitochondria and is found in most plants and animals (Tan D-X et al. 2013, Mitochondria and chloroplasts as the original sites of melatonin synthesis: a hypothesis related to melatonin's primary function and evolution in eukaryotes. J Pineal Res 54: 127 – 138). So, there is likely to be endogenous melatonin in the cotton seed. Melatonin is extremely efficient antioxidant (Tan DX, Manchester LC, Terron MP, Flores LJ, Reiter RJ. One molecule, many derivatives: a never-ending interaction of melatonin with reactive oxygen and nitrogen species? J Pineal Res 2007; 42: 28 – 42; reference 90). Line 64: Melatonin is closely related to IAA (indole -3-acetic acid) by structure and so are their metabolic pathways (see, for instance, Chapter 2, Auxin Biosynthesis and Catabolism, Yangbin Gao and Yunde Zhao, in Auxin and its Role in Plant Development, 2014, Eds. Zazimalova E, Petrasek J, Benkova E, Springer or Arnao MB and Hernandez-Ruiz J, 2018, Melatonin and its relationship to plant hormones, Annals of Botany 121: 195–207).

**Response:** We have made correction according to the Reviewer’s comments. We have supplemented the relevant reference.

**Methods**

19. How was the salt stress level of 150 mM chosen? While the difference between salt stressed and control germination rates show clear significance, the effects of different melatonin concentration are very similar (especially comparing 10 and 20 μM data) in Fig. 1. Could greater saline stress give more distinct results?

**Response:** We have made correction according to the Reviewer’s comments. We have screened the salt concentration through pre-experiment. The data have been published in the journal cotton, but not yet in print.

20. I am also puzzled by the assignment of the significant difference (or lack there of) in some of the figures: for instance, in Fig. 2, at 6 d, MT50 and MT100 are surely not significantly different (but assigned letters “b” and “d”), while MT10 and MT20 are significantly different (but both assigned same letter “e”). There seem to be significance assignment problems in most of the figures (I am assuming that decreasing averages are assigned consecutive letters of the alphabet, if they are significantly different, depending on their standard deviations)

**Response:** We have made correction according to the Reviewer’s comments. We recheck the statistical analysis and redo the statistical analysis.

21. The units in some of the figures are not clear. Fig. 2: what does “mmol/g prot” mean? Fig. 3: microSiemens/cm? Fig. 4B: why not use microg? Fig. 4C: why is “fresh weight” added here – what about the Fig. 4A and B? Fig. 5A, B and C: why different units in A? Same type of units should be used for measurement of various substances, so the amounts can be compared (for instance amounts of proline and soluble sugars or Na, Cl and K).

**Response:** We have made correction according to the Reviewer’s comments.

22. It would be informative to measure endogenous melatonin content of the germinating seeds in control and under saline stress.

**Response:** Thank you for your suggestion. We will elaborate on the endogenous melatonin content in another manuscript.

**Results**

23. While there are problems with the significance of the differences, the consistent trend of MT20 data does suggest, that this melatonin concentration is the most supportive one for germination in the face of saline stress.

**Response:** We have made correction according to the Reviewer’s comments.

**Discussion**

24. The authors should comment on: (1) The changes of the measured substances in the control and salt stressed seeds with time, which makes evaluating the role of melatonin more difficult, (2) Why is MT20 the most beneficial medium – the endogenous melatonin concentration might be helpful there.

**Response:** We have made correction according to the Reviewer’s comments.

(1) The change of the measured substances in the control and salt stressed seeds with time was added in discussion.
(2) In the paper, the effect of exogenous application melatonin of 20 μM all exhibited a beneficial effect in inhibiting H_2_O_2_, EL, MDA accumulation and promoting accumulation of proline, soluble sugar, protein as well as maintaining ion balance under salt stress condition, showing MT20 is the most beneficial medium in promoting seed germination.
(3) As for endogenous melatonin concentration, we will plan to do it next. Thanks for the Reviewer’s suggestions.

**Reference**

25. Reference 20 and 32 seem to be the same paper.

**Response:** We have made correction according to the Reviewer’s comments and delete the reference of 32.

Special thanks to you for your good comments.

Sincerely yours,
Zhiying Bai
Email: zhiyingbai@126.com
