# Supplementary material for: Exogenous melatonin promotes seed germination and osmotic regulation under salt stress in cotton (Gossypium hirsutum L.)
Source: PLoS One. 2020 Jan 31;15(1):e0228241. doi: 10.1371/journal.pone.0228241 (PMC6994006; doi:10.1371/journal.pone.0228241)
Supplement: Attachment — Submitted filename: Response to Reviewers.docx [file pone.0228241.s004.docx]

Dear Sergey Shabala

On behalf of my co-authors, we thank you very much for giving us an opportunity to revise our manuscript again, we appreciate editor and Reviewers very much for their positive and constructive comments and suggestions on our manuscript entitled “Exogenous melatonin promotes seed germination and osmotic regulation under salt stress in cotton (*Gossypium hirsutum* L.) (submitted as PONE-D-19-18506)”. Those comments are all valuable and very helpful for revising and improving our paper, as well as the important guiding significance to us researches. We have studied comments carefully and have made correction which we hope meet with approval. Revised portion are marked in red in the paper.

The main corrections in the paper and the responds to the Reviewer’s comments are as flowing:

Responds to the Reviewer’s comments:

**Reviewer #1**

**Few more comments**

1. Please double check the English of the newly added text.

**Response:** We have made correction according to the Reviewer’s comments. Our manuscript has been carefully edited by a native English-speaking editor of MogoEdit, and the grammar, spelling, and punctuation have been verified and corrected where needed.

2. Add a vertical dash line between the dataset of different germination time points to indicate that the statistical analysis was separately done in each germination time point group

**Response:** We have made correction according to the Reviewer’s comments. We have added a vertical dash line between the data at of different germination time points. Thank you for your suggestion.

3. Maathuis, 2014, Sodium in plants: perception, signaling, and regulation of sodium fluxes. Journal of experimental Botany.

**Response:** We have made correction according to the Reviewer’s comments. We have added reference.

**Reviewer #2**

1. The manuscript is improved, but still marginal. The assignment of significance in many of the figures is still a problem: for instance in Fig. 1 the control at 2 days is surely significantly different from the control at 6 days, yet they are both assigned letter “a”! The “c” and “d” are not significantly different! You do want to compare amounts at different germination times.

**Response:** We have compared amounts at different treatments according to the Reviewer’s comments.

As for the assignment of significance in many of the figures, the meaning of the letter (a, b, c, d) is explained as follows:

In Fig. 1, firstly, we used SPSS software to conduct variance analysis on the data of different treatments (Control; NaCl; MT_10_+NaCl; MT_20_+NaCl; MT_50_+NaCl; MT_100_+NaCl) at 2 d, different letters (a, b, c, d) appeared in 2 d group. Then we used SPSS software to conduct variance analysis on the data of different treatments (Control; NaCl; MT_10_+NaCl; MT_20_+NaCl; MT_50_+NaCl; MT_100_+NaCl) on 4 d or 6 d respectively, different letters (a, b, c, d) also appeared in 4 d or 6 d group. So the control at 2 days is significantly different from the control at 6 days, yet they are both assigned letter “a”.

In addition, data from other figures and tables are analyzed in the same way.

2. The measurement of the melatonin content in the control and in the seeds under different treatments would make it easier to formulate the mechanism of action

**Response:** We have made correction according to the Reviewer’s comments. We have supplemented the measurement of the melatonin content in the control and in the seeds under different treatments

3. The germination rate increased over 6 days in the control. However, the various measured substances changed over this time. These trends need to be compared and discussed for different treatments in detail. For instance: hydrogen peroxide is quite high in the control at day 2, drops drastically in day 4 and rises again in day 6. In the salinity treatment the amount at day 6 is less than the control at day 2, but the germination rate is significantly less than the control at any time. The MT+50, MT+100 treatments decreased the hydrogen peroxide substantically at day 4 and 6, but have not improved the germination rate. Consequently, the amount of hydrogen peroxide may not be particularly important variable, or the seed germination process may be quite tolerance to it. The proline content drops in the control over time, but increases in saline treatment on day 2, but then drops to similar level as in control on day 4 and 6. MT+20 increases proline levels at all time, contributing to better osmotic state of the seed. It is interesting that increased melatonin concentration does not lead to more proline.

**Response:** We have made correction according to the Reviewer’s comments. The detailed changes can be seen in lines 474-499 of the discussion.

4. Discussion: All measured substances should be considered and compared to the time variation in the control and the saline stressed plants. So, the results need to be discussed in much greater detail with referances to revelant tables and figures (e.g. lines 426-433). The general problems with salinity (lines 418-421) and the properties of melatonin (lines 435-439) were already described in the introduction. The authors do not speculate why there is an optimal melatonin concentration. Reactive oxygen species (ROS) are also involved in signalling sequences. It is possible that too much melatonin disrupts these sequences in the germination seed (however, this is not the case with hydrogen peroxide, which is higher at MT+50 and MT+100).

**Response:** We have made correction according to the Reviewer’s comments.The detailed changes can be seen in 502-525 of the discussion.

Special thanks to you for your good comments.

Sincerely yours,
Zhiying Bai
Email: zhiyingbai@126.com
