# Supplementary material for: Exogenous melatonin promotes seed germination and osmotic regulation under salt stress in cotton (Gossypium hirsutum L.)
Source: PLoS One. 2020 Jan 31;15(1):e0228241. doi: 10.1371/journal.pone.0228241 (PMC6994006; doi:10.1371/journal.pone.0228241)
Supplement: Attachment — Submitted filename: Response to Reviewers.docx [file pone.0228241.s005.docx]

Dear Sergey Shabala

On behalf of my co-authors, we thank you very much for your feedback and the opportunity to revise our manuscript again. We also thank the Reviewers very much for their positive and constructive comments and suggestions on our manuscript entitled “Exogenous melatonin promotes seed germination and osmotic regulation under salt stress in cotton (*Gossypium hirsutum* L.) (submitted as PONE-D-19-18506).” These comments were all valuable and very helpful in our efforts to improve our paper and also provided significant guidance in our research. We have considered the comments carefully and have made correction that we hope will meet with approval. The revised portions are marked in red in the paper.

Below, the main corrections to the paper and responses to the Reviewer’s comments are provided.

Responses to the Reviewer’s comments:

**Reviewer #2:** The measurements of melatonin content add to the information on the melatonin effect on the germination seeds-important increased of the dataset.

However, the results are still not discussed clearly and logically.

For instance: The germination rate increases with time. Similar pattern is observed in salt-stressed seeds, but with reduced rates. MT20 improves the rates at all times, but not up to control level. MT50 and 100 essentially leave the salt-stressed rate unchanged (Fig. 1). Melatonin levels decrease in the control (Fig. 2). There is not sufficient data to decide if the seeds make less melatonin or equal (or greater) amounts, which get consumed by the antioxidant action. Lines 253-255: therefore it is not clear whether salinity stress “inhibits” melatonin synthesis. As there is less reactive oxygen species with more added melatonin, it is more likely that it is consumed by antioxidant action of the melatonin.

It is good to know that increased external melatonin translates to increased internal melatonin (Fig. 2). However, this increase in internal melatonin does not help to keep the germination rates up in case of MT50 and 100.

The M20 medium is most efficient in increasing the concentration of proline, soluble sugar and soluble protein and these substances need to be greater than in the control to improve osmotic balance in salt stressed seeds (Fig. 5). However, even in the control these substances vary with time.
In the Discussion, the authors need to distinguish between regulatory changes in the seed upon exposure to saline stress (such as increase in proline) or a change due to loss of control (such as increase in Na+).

So, the data still needs to be discussed in more consistent and logical manner.

**Response:** We have made corrections according to the Reviewer’s comments. The detailed changes can be seen in lines 440-451, 453-459, 475-477, 48-484, and 535-540 in the Discussion. Additionally, two new references have been added as support.

Special thanks to you for your good comments.

Sincerely yours,
Zhiying Bai
Email: zhiyingbai@126.com
